# The Dual Challenges of Generality and Specificity When Developing Environmental DNA Markers for Species and Subspecies of *Oncorhynchus*

**DOI:** 10.1371/journal.pone.0142008

**Published:** 2015-11-04

**Authors:** Taylor M. Wilcox, Kellie J. Carim, Kevin S. McKelvey, Michael K. Young, Michael K. Schwartz

**Affiliations:** 1 United States Department of Agriculture, Forest Service, National Genomics Center for Wildlife and Fish Conservation, Rocky Mountain Research Station, Missoula, Montana, United States of America; 2 University of Montana, Division of Biological Sciences, 32 Campus Dr., Missoula, Montana, United States of America; Central Michigan University, UNITED STATES

## Abstract

Environmental DNA (eDNA) sampling is a powerful tool for detecting invasive and native aquatic species. Often, species of conservation interest co-occur with other, closely related taxa. Here, we developed qPCR (quantitative PCR) markers which distinguish westslope cutthroat trout (*Oncorhynchus clarkii lewsi*), Yellowstone cutthroat trout (*O*. *clarkii bouvieri*), and rainbow trout (*O*. *mykiss*), which are of conservation interest both as native species and as invasive species across each other’s native ranges. We found that local polymorphisms within westslope cutthroat trout and rainbow trout posed a challenge to designing assays that are generally applicable across the range of these widely-distributed species. Further, poorly-resolved taxonomies of Yellowstone cutthroat trout and Bonneville cutthroat trout (*O*. *c*. *utah*) prevented design of an assay that distinguishes these recognized taxa. The issues of intraspecific polymorphism and unresolved taxonomy for eDNA assay design addressed in this study are likely to be general problems for closely-related taxa. Prior to field application, we recommend that future studies sample populations and test assays more broadly than has been typical of published eDNA assays to date.

## Introduction

Environmental DNA (eDNA) sampling is the search for genetic material in the environment (e.g., water or soil) to infer species presence [1]. This approach is particularly well suited to detecting aquatic species when they are rare, such as small populations of endangered native species or new invasions of introduced species (e.g., recent reviews include [2–4]). Many recent applications of eDNA sampling have focused on one to several taxa, using cost-effective species-diagnostic mitochondrial markers (i.e., species-specific PCR; e.g., [5,6]). The ideal taxon-specific marker will have both high specificity (amplifying only the DNA of the target taxon) and broad generality (amplifying the DNA of all populations of the target taxon across its range). Attaining both goals, however, can be challenging [7]. Achieving specificity, for example, has been problematic when attempting to distinguish among closely related taxa. For example, Wilcox et al. [7] found that insufficient specificity in a quantitative PCR (qPCR) assay could result in reduced detectability for the target species in mixed samples of closely related chars (salmonid fishes in the genus *Salvelinus*). Conversely, Fukumoto et al. [8] were unable to reliably distinguish the eDNA from two closely related and hybridizing salamanders (*Andrias* spp.) using qPCR, and resorted to additional sequencing to confirm species identity. Alternatively, achieving generality in an eDNA assay is difficult for taxa with substantial phylogenetic structure because some populations or clades may exhibit polymorphisms that reduce assay sensitivity. For example, Goldberg et al. [9] designed species-specific hydrolysis markers (TaqMan) to detect invasive New Zealand mudsnail (*Potamopyrgus antipodarum*). After developing their assay, a new haplotype with a polymorphism within the locus of one of the assay primers was discovered. They confirmed that this polymorphism did not affect assay sensitivity, but point out that if this polymorphism had been on the 3’ end of an amplification primer there could have been false negative results from environmental samples with low quantities of DNA. Detecting these polymorphisms requires examination of samples of a target taxon from across its range (e.g., [10]).

**Fig 1:**
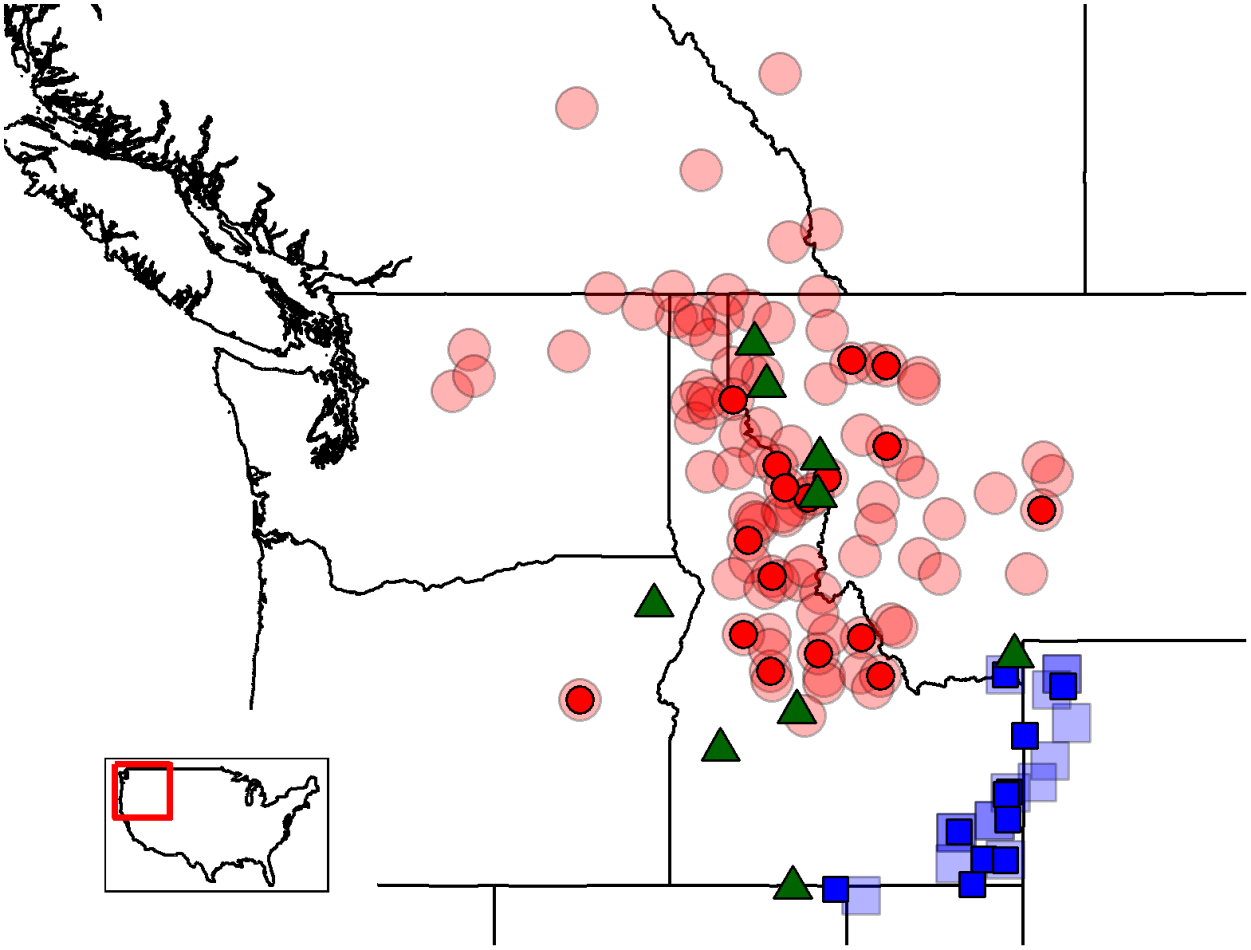
Environmental DNA marker development may be difficult or impossible when the target taxon’s taxonomy is unresolved or has poor concordance with true phylogeny. Colored boxes show recognized taxa and black lines show the phylogenetic relationships among populations. (A) Taxon X includes multiple, divergent lineages. It is important that all of these lineages are sampled during marker validation to insure intraspecific generality. Even extensive sampling of a single lineage (red circle) is not sufficient and will lead to ascertainment bias and low marker generality. (B) Recognized taxa X and Y are not monophyletic (i.e., they are both paraphyletic groups), which may make distinguishing between them using sequence data impossible. (C) Recognized taxon X includes divergent lineages (polyphyly), but some of those lineages may also not be distinguishable from recognized taxon Y.

Additional challenges in building eDNA assays arise when there are conflicts between taxonomy and phylogenetic relationships, or when phylogenetic relationships are unresolved. Developing an assay with generality within a taxon may be difficult or impossible when divergent lineages are assigned to a single taxon (Fig 1A). Conversely, there may be few or no diagnostic loci that distinguish among taxa lacking phylogenetic divergence (e.g., paraphyly; Fig 1B). These scenarios are not mutually exclusive—some taxa may comprise markedly divergent lineages (polyphyly), yet also lack divergence from other named taxa (Fig 1C).

We confronted these issues in developing qPCR assays for eDNA detection of three salmonid taxa native to western North America: rainbow trout (RBT; *Oncorhynchus mykiss*) and two subspecies of cutthroat trout (*O*. *clarkii*), westslope cutthroat trout (WCT; *O*. *c*. *lewisi*) and Yellowstone cutthroat trout (YCT; *O*. *c*. *bouvieri*). Rainbow trout are the sister species to cutthroat trout (genetic distance = 7.3–8.7% in *NADH2*), whereas WCT and YCT (genetic distance = 2.7%; [11]) are subspecies both found in interior western North America [12]. In portions of the Columbia River basin, RBT and WCT naturally co-occur, but introductions of all three taxa outside their historical ranges have led to far greater recent sympatry [12]. Despite global increases in their distributions, the distribution of native populations of these three taxa have declined [13–15]. Consequently, identifying the current distributions of both native and introduced populations is a priority for management agencies [13,16,17]. Environmental DNA sampling could be a useful tool to detect the presence of each taxon, whether at the leading edge of an invasion or a remnant native population. Because RBT, WCT, and YCT are closely related and commonly co-occur, highly specific eDNA assays are critical for robust detection, particularly where the target taxon is rare and one or both of the others are common [7]. To address these challenges, we examined DNA sequences and samples from across the historical ranges of the two cutthroat trout subspecies and from an array of native populations and hatchery stocks of rainbow trout, and used an automated pipeline to generate and test candidate assays for each taxa.

## Methods

### Sequence data for assay development

**Fig 2:**
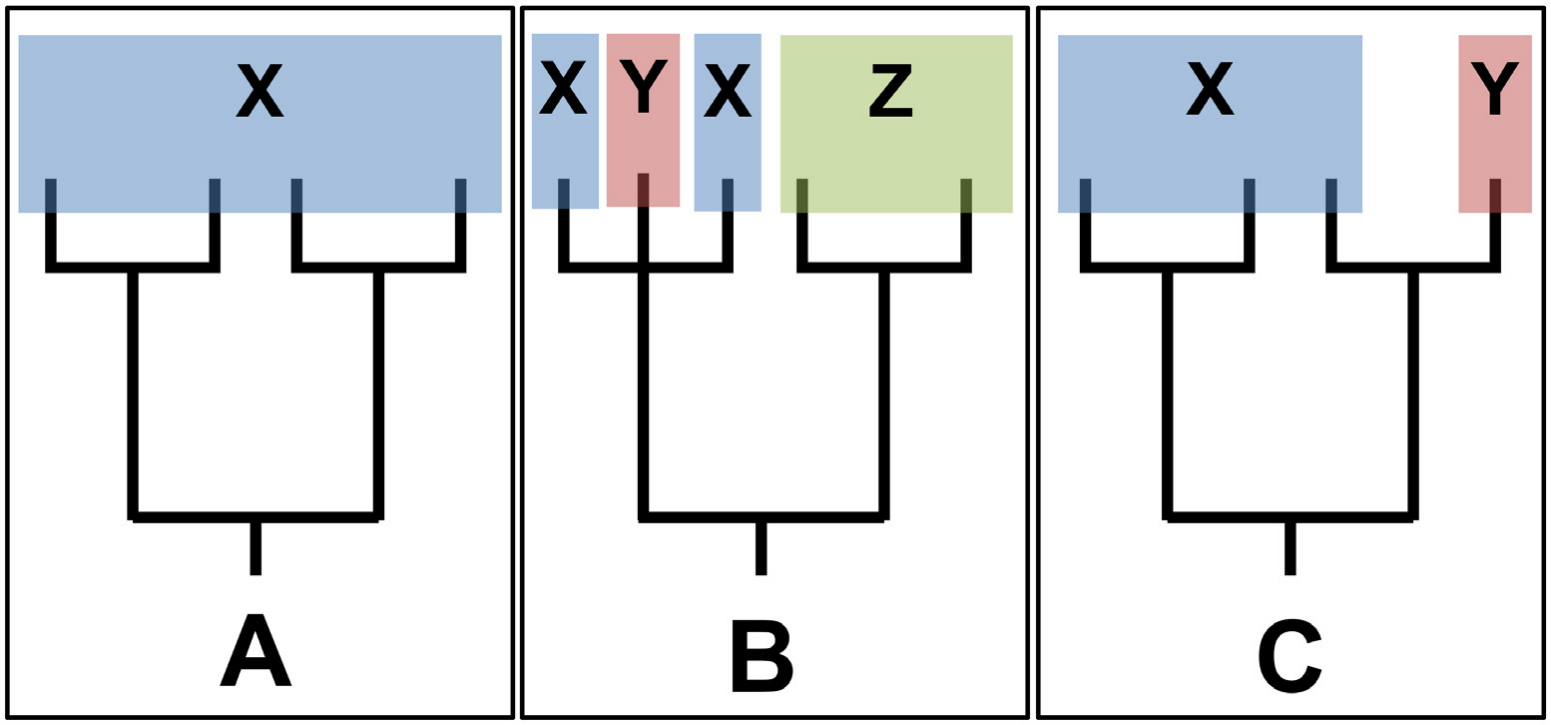
Source locations for rainbow trout (green triangles), westslope cutthroat trout (red circles), and Yellowstone/Bonneville cutthroat trout (both subspecies indicated with blue squares) sampled from the wild for assay development (large, light-colored shapes) and for assay testing (small, dark-colored shapes). The source locations for one rainbow trout sample obtained from Alaska (USA), all hatchery-derived fish, and the Pacific salmon congeners used in assay testing are not shown here, but are listed in Table 1.

We assembled sequence data for our target taxa and three other congeners—Chinook salmon (*O*. *tshawytscha*), coho salmon (*O*. *kisutch*), and sockeye salmon (*O*. *nerka*)–to design qPCR assays in the *NADH* region of the mitogenome. We used *NADH* because it tends to show relatively high sequence divergence and is one of the most commonly archived mitochondrial sequences for salmonids. For YCT, we obtained sequences from a 3,483-bp region of the *NADH* subunits 1 and 2 for 17 individuals from across its range ([18]; GenBank accession numbers: EU186781.1 –EU186797.1; Table 1 and Fig 2). We obtained these same gene regions from whole mitogenomes for five RBT from Pacific North America (GenBank accession numbers: DQ288268.1 –DQ288271.1, L29771.1) and each of the Pacific salmon congeners listed above (GenBank accession numbers: AF392054.1 and NC_002980.1 for Chinook, EF126369.1 for coho, and EF055889.1 for sockeye). We used previously unpublished sequences from 96 WCT from across the species’ range (S1 Text). All tissue samples used in this study were stored in ethanol, lysis buffer, or dried on chromatography paper. All samples used in this study were provided by collaborators from previous studies conducted under appropriate scientific sampling permits or from collections under Montana Fish, Wildlife and Parks Scientific Collectors Permits 12–2001, 14–2001, and 19a-2009 issued to M. K. Young (Table 1, Fig 2). Sampling sites were accessed via public land and did not require special permission. Sampling of protected species was allowed under U.S. Fish and Wildlife Service Federal Fish and Wildlife Permit TE220826-0 issued to M. K. Young. Animals were captured via backpack electrofishing, a small fin tissue sample was collected, and then the animal was released at the place of capture in accordance with a protocol approved under these scientific sampling permits. DNA was extracted from these samples using QIAGEN DNeasy Blood and Tissue Kit following the manufacturer’s protocol.

**Table 1:** Source populations for fish used in assay design and testing.

Species	Souce	Purpose
Yellowstone cutthroat trout	Clear Cr (Idaho, USA)	Sequence data; assay testing
	Barnes Cr (Idaho, USA)	Sequence data; assay testing
	Goose Cr (Nevada, USA)	Sequence data; assay testing
	Cottonwood Cr, (Idaho, USA)	Sequence data; assay testing
	Upper Blackfoot R (Montana, USA)	Sequence data; assay testing
	Badger Cr (Idaho, USA)	Sequence data; assay testing
	Yellowstone L (Wyoming, USA)	Sequence data; assay testing
Bonneville cutthroat trout	Bear L (Utah, USA)	Sequence data; assay testing
	Bear R (Idaho, USA)	Sequence data; assay testing
	Glenwood FH (Utah, USA)*	Sequence data; assay testing
	Harkness Cr (Idaho, USA)	Sequence data; assay testing
Rainbow trout	Skookumchuck R FH (Washington, USA)*	Sequence data
	Gulkana R (Alaska, USA)*	Sequence data
	Dworshak FH (Idaho, USA)*	Sequence data
	Ennis FH—Arlee (Montana, USA)*	Assay testing
	Ennis FH—Eagle L (Montana, USA)*	Assay testing
	Ennis FH—Fish L (Montana, USA)*	Assay testing
	Ennis FH—Shasta (Montana, USA)*	Assay testing
	Ennis FH—McConaughy (Montana, USA)*	Assay testing
	Dry Cr (Idaho, USA)	Assay testing
	Sawtooth FH (Idaho, USA)*	Assay testing
	Shack Cr (Idaho, USA)	Assay testing
	Wallowa FH (Idaho, USA)*	Assay testing
	Bobtail Cr (Montana, USA)	Assay testing
	Silver Butte Fisher R (Montana, USA)	Assay testing
	E.F. Lolo Cr (Montana, USA)	Assay testing
	McCormick Cr (Montana, USA)	Assay testing
	Red Canyon (Montana, USA)	Assay testing
Westslope cutthroat trout	John Day R (Oregon, USA)	Sequence data; assay testing
	NF Elkhorn Cr (Idaho, USA)	Sequence data; assay testing
	Split Cr (Idaho, USA)	Sequence data; assay testing
	Withington Cr (Idaho, USA)	Sequence data; assay testing
	Duck Cr (Idaho, USA)	Sequence data; assay testing
	Heller Cr (Idaho, USA)	Sequence data; assay testing
	Rampike Cr (Idaho, USA)	Sequence data; assay testing
	Ditch Cr (Idaho, USA)	Sequence data; assay testing
	Crooked Fork Cr (Idaho, USA)	Sequence data; assay testing
	Pete King Cr (Idaho, USA)	Sequence data; assay testing
	Osier Cr (Idaho, USA)	Sequence data; assay testing
	Albert Cr (Montana, USA)	Sequence data; assay testing
	Fourmile Cr (Montana, USA)	Sequence data; assay testing
	Youngs Cr (Montana, USA)	Sequence data; assay testing
	Wounded Buck Cr (Montana, USA)	Sequence data; assay testing
	Twentyfivemile Cr (Montana, USA)	Sequence data; assay testing
	Flat Cr (Idaho, USA)	Sequence data
	Meadow Cr (Idaho, USA)	Sequence data
	French Cr (Idaho, USA)	Sequence data
	Baldy Cr (Idaho, USA)	Sequence data
	Twisp R (Washington, USA)	Sequence data
	Buck Cr (Washington, USA)	Sequence data
	American R (Idaho, USA)	Sequence data
	Falls Cr (Washington, USA)	Sequence data
	Hungery Cr (Idaho, USA)	Sequence data
	Cayuse Cr (Idaho, USA)	Sequence data
	Indian Grave Cr (Idaho, USA)	Sequence data
	Gravey Cr (Idaho, USA)	Sequence data
	Beaver Cr (Montana, USA)	Sequence data
	Moose Cr (Montana, USA)	Sequence data
	Deer Cr (Montana, USA)	Sequence data
	EF Bull R (Montana, USA)	Sequence data
	Ketchikan Cr (Montana, USA)	Sequence data
	McGuire Cr (Montana, USA)	Sequence data
	Martin Cr (Montana, USA)	Sequence data
	McCabe Cr (Montana, USA)	Sequence data
	Miller Cr (Montana, USA)	Sequence data
	Norton Cr (Montana, USA)	Sequence data
	Ontario Cr (Montana, USA)	Sequence data
	North Cr (Montana, USA)	Sequence data
	Tyler Cr (Montana, USA)	Sequence data
	Wilkes Cr (Montana, USA)	Sequence data
	Canuck Cr (Idaho, USA)	Sequence data
	Ball Cr (Idaho, USA)	Sequence data
	West Gold Cr (Idaho, USA)	Sequence data
	Beaver Cr (Idaho, USA)	Sequence data
	Mokins Cr (Idaho, USA)	Sequence data
	NF St. Joe R (Idaho, USA)	Sequence data
	Skin Cr (Idaho, USA)	Sequence data
	Slowey Cr (Montana, USA)	Sequence data
	Dry Wolf Cr (Montana, USA)	Sequence data
	Tributary of Armstrong Cr (Idaho, USA)	Sequence data
	Bad Luck Cr (Idaho, USA)	Sequence data
	Cedar Cr (Washington, USA)	Sequence data
	Ninemile Cr (Washington, USA)	Sequence data
	Scotchman Gulch (Montana, USA)	Sequence data
	Kraft Cr (Montana, USA)	Sequence data
	Leiberg Cr (Idaho, USA)	Sequence data
	N. Grouse Cr (Idaho, USA)	Sequence data
	SF Red R (Idaho, USA)	Sequence data
	Yoosa Cr (Idaho, USA)	Sequence data
	Ross Cr (Montana, USA)	Sequence data
	EF Emerald Cr (Idaho, USA)	Sequence data
	Bitter Cr (B.C., Canada)	Sequence data
	Blairmore Cr (B.C., Canada)	Sequence data
	Crazy Cr (B.C., Canada)	Sequence data
	Hartley Cr (B.C., Canada)	Sequence data
	Monk Cr (B.C., Canada)	Sequence data
	Pack R (Idaho, USA)	Sequence data
	Sheep Cr (Montana, USA)	Sequence data
	Toby Cr (B.C., Canada)	Sequence data
	Truman Cr (Montana, USA)	Sequence data
	Twin Cr (Idaho, USA)	Sequence data
	Waiparous Cr (B.C., Canada)	Sequence data
	Werner Cr (Montana, USA)	Sequence data
	Bostwick Cr (Montana, USA)	Sequence data
	Avalanche Cr (Montana, USA)	Sequence data
	Fish Cr (Montana, USA)	Sequence data
	NF Dupuyer Cr (Montana, USA)	Sequence data
	Sawmill Cr (Montana, USA)	Sequence data
	Fourmile Cr (Montana, USA)	Sequence data
	NF Teton R (Montana, USA)	Sequence data
	Thayer Cr (Montana, USA)	Sequence data
	Buffalo Cr (Montana, USA)	Sequence data
	Bluff Cr (Idaho, USA)	Sequence data
	Trail Cr (Idaho, USA)	Sequence data
	Floodwood Cr (Idaho, USA)	Sequence data
	Jacobs Ladder Cr (Idaho, USA)	Sequence data
	Blackbird Cr (Idaho, USA)	Sequence data
	Mill Cr (Idaho, USA)	Sequence data
	Morse Cr (Idaho, USA)	Sequence data
	MF Little Timber Cr (Idaho, USA)	Sequence data
	Big Cr (Idaho, USA)	Sequence data
	Boundary Cr (Idaho, USA)	Sequence data
	Colson Cr (Idaho, USA)	Sequence data
	Warm Springs Cr (Idaho, USA)	Sequence data
Chinook salmon	SF Salmon R (Idaho, USA)*	Assay testing
	Pahsimeroi R (Idaho, USA)*	Assay testing
	Clearwater R (Idaho, USA)*	Assay testing
Coho salmon	Nez Perce Tribal FH (Idaho, USA)*	Assay testing
Sockeye salmon	Redfish L (Idaho, USA)*	Assay testing

Locations marked with an asterisk are not shown in Fig 2. Sequencing; sequences from fish were used for initial assay design, Assay testing; extracted tissue samples from fish were used for assay testing.

### Primer development

We used the *DECIPHER* package [19] in *R* v. 3.0.1 [20] to generate candidate primer sets for the *NADH* gene region for each species using the sequence data from above. We visually compared alignments of these candidate primers with consensus sequences of all *Oncorhynchu*s species in *MEGA5* [21], and adjusted primer length to optimize annealing temperature in *Primer Express* v. 3 (Life Technologies).

Candidate primer sets (*n* = 3, 4, and 5 for WCT, YCT, and RBT respectively) were tested against extracted DNA from tissues of three individuals of each *Oncorhynchus* taxon in 20-μl reactions composed of 0.1 ng of DNA (extracted from tissue using QIAGEN DNEasy Blood and Tissue Kit following the manufacturer’s protocol and quantified on a NanoPhotometer; IMPLEN), 1X concentration of SYBR Green PCR Mastermix (Life Technologies) and each primer at 150 nM. We used cycling conditions of 95°C/10 min [95°C/15 s, 60°C/60 s] × 45 cycles on a StepOne Plus Real-time PCR Instrument (Life Technologies), followed by a melt curve from 65°C to 95°C in 0.3°C increments (to test for primer dimer formation). Candidate primer sets for all three target species were tested both with and without an induced mismatch (single base pair mismatch with both target and non-target sequences) in the reverse primer six nucleotides from the 3' end to increase target specificity (i.e., reduce hybridization with non-target sequences [22]). To determine if this induced mismatch influenced sensitivity, we compared amplification curves of primer sets both with and without the mismatch. In all cases, there was no detectable difference in cycle threshold (C_t_) when using DNA from the target taxon, but an increased delay in amplification when using DNA from non-target taxa, so we used primer sets with the induced mismatch for probe design.

### Probe development and assay optimization

We used *PrimerExpress* 3.0 (Life Technologies) to design eight hydrolysis probes (TaqMan-MGB probes) for the seven most target-specific primer sets (*n* = 2, 2, and 4 probes and 2, 2, and 3 primer sets for WCT, YCT, and RBT respectively). We optimized primer concentrations for primer-limiting reactions to increase assay specificity and ease of future multiplexing. This was done by independently varying final primer concentrations to 100, 300, 600, and 900 nM (*n* = 16 combinations; probe concentration held at 250 nM). We used the lowest primer concentrations that also resulted in the lowest C_t_ value while maintaining high end-point fluorescence relative to the highest primer concentration for each assay for further testing. Screening of the assays with probes were done in 15-μl reaction volumes composed of ~ 0.1 ng of DNA, a final concentration of 1X Environmental Mastermix 2.0 (Life Technologies), and optimized concentrations of the primers and probe (Table 2) on a StepOne Plus Real-time PCR Instrument (Life Technologies) using the same cycling conditions as above (except without a final melt curve step).

**Table 2:** Sequences for each of the validated taxon-specific qPCR assays.

Species	Oligo	Sequence	Rxn [] (nM)	Amplicon ln
westslope cutthroat	F	5’-CCTAAAACTATTTATTAAAGAACCAGTTCG-3’	100	88
	R	5’-AAGTGTAAGGGCGAGTCTRGGG-3’	900	
	P	6FAM-5’-CCACCTCCTCTCCCT-3’ -MGBNFQ	250	
Yellowstone cutthroat	F	5’-CGACCTTCCACCTCCTCC-3’	600	152
	R	5’-AGCTAGACTGGATAGCTCAAGC-3’	900	
	P	6FAM-5’-CTCGCCACACCTATACT-3’ -MGBNFQ	250	
rainbow trout	F	5’-AGTCTCTCCCTGTATATCGTC-3’	300	102
	R	5’-GATTTAGTTCATGAAGTTGCGTGAGTA-3’	600	
	P	6FAM-5’-CCAACAACTCTTTAACCATC-3’ -MGBNFQ	250	

Target species, oligonucleotide (forward primer; F, reverse primer R, probe; P), oligonucleotide sequence, and optimized reaction concentration of the oligonucleotide (nM), and amplicon length (bp). The induced mismatch (mismatch between both target and non-target taxa to increase specificity) in each reverse primer is underlined.

We screened these assays against the same individuals above for specificity, as well as samples of three commonly sympatric salmonids: brook trout (*Salvelinus fontinalis*), bull trout (*Salvelinus confluentu*s), and brown trout (*Salmo trutta*; Morrell Creek, Montana, USA; N 47.1779 W -113.4699) to select assays for final screening. All assays that did not amplify DNA from non-target species were tested for generality by screening against tissue samples from across the northwestern U.S. range of each of the three target taxa (*n* = 30 each), including collections from seven RBT hatchery strains and samples covering most of the range of each subspecies of cutthroat trout (Table 1 and Fig 2). The panel for YCT also included 11 individuals of the closely related Bonneville cutthroat trout (BCT; *O*. *c*. *utah*; [11]). We also tested for non-target template competition [7] by amplifying mixed samples in which the DNA of a target taxon made up 1% and DNA of the other five *Oncorhynchus* made up 99% of the sample (i.e., approximately 0.001 ng of target DNA and 0.099 ng of non-target DNA).

### Assay sensitivity

To determine the amplification efficiency and limit of detection for a final set of three taxon-specific assays, we performed standard curve experiments for each. For each assay, a synthetic template was prepared by ordering a synthetic gene from Integrated DNA Technologies that included the target amplicon sequence. The lyophilized gene was resuspended in sterile TE, linearized with a *Pvu1* restriction digest, purified, and quantified on a Quibit 2.0 fluorometer (ThermoFisher Scientific; see [23] for details). From this stock, a five-level dilution series (6 250, 1 250, 250, 50, and 10 copies/4 μl) was prepared in sterile TE. We ran six replicates of each dilution series for each assay to determine the standard curve slope (amplification efficiency) and limit of detection (lowest concentration with >95% amplification success [24]).

## Results

We optimized a final set of three assays—one for each target taxon (Table 2). These assays had no amplification of non-target DNA, except that the YCT assay also amplified DNA from closely-related Bonneville cutthroat trout (Table 3). Assays were not influenced by non-target template competition (i.e., all mixed samples had amplification of the target DNA). However, the RBT and WCT assays did have reduced amplification efficiency or failed amplification for some individuals within each taxon (Table 3).

**Table 3:** Summary of results of the validated taxon-specific qPCR assays.

Assay	Problematic polymorphism
Yellowstone cutthroat	Bonneville cutthroat trout also amplify
westslope cutthroat	Rare polymorphism caused some Youngs Cr individuals not to amplify
rainbow trout	Delayed amplification for individuals from Eagle Lake (*O*. *mykiss aquilarium*)

Except for the noted problematic polymorphisms, each assay was taxon-specific and amplified all individuals of the target taxon with similar efficiency.

### Yellowstone cutthroat trout

The YCT panel of 30 fish for assay testing included both YCT and BCT individuals, which are recognized as separate subspecies, but which have little genetic divergence across much of their range [11]. DNA from all of these individuals amplified, but amplification efficiency was substantially decreased (~ 10 C_t_ amplification curve delay) for BCT individuals from Harkness Creek and Glenwood Fish Hatchery (*n* = 3 and 2 individuals, respectively; six BCT individuals from Bear Lake and Bear River all amplified efficiently). The mtDNA haplotypes of individuals sampled from these Harkness Creek and Glenwood Fish Hatchery populations have three base-pair polymorphisms within the reverse primer binding region of our assay (7, 16, and 19 bp from the 3’ end of the primer [18]). These base-pair differences reduce primer hybridization, resulting in the substantially increased C_t_ for these individuals. Based on the standard curve experiments, this assay had an amplification efficiency of 95.5% (standard curve y-intercept = 43.4, *r* ^*2*^ = 0.965) and limit of detection of 10 mtDNA copies/rxn.

### Westslope cutthroat trout

The marker for WCT was designed with a degenerate base in the reverse primer to account for a known intraspecific polymorphism. All of the 30 individuals in the screening panel for this assay amplified with the same efficiency. However, after assay design, we discovered another polymorphism within the probe-binding region of this assay in a sequence from a single fish captured in Youngs Creek (tributary to the South Fork Flathead River, Montana, USA) which we had not previously noted. We tested the assay against ten individuals from this population. Six individuals amplified as expected, but we observed complete amplification failure of the remaining four fish. There was 100% amplification success in an additional 20 fish screened from within the same river basin (ten each from two other streams < 100 km downstream in the Flathead River basin, Montana, USA). Based on the standard curve experiments, this assay had an amplification efficiency of 96.3% (standard curve y-intercept = 40.4, *r* ^*2*^ = 0.971) and limit of detection of 50 mtDNA copies/rxn.

### Rainbow trout

The marker for RBT amplified all 30 individuals in the screening panel; however, the two individuals from the Eagle Lake hatchery strain (Table 1) had reduced amplification efficiency (~ 2 C_t_ delay in amplification). This commonly stocked strain is sourced from an isolated lake population and may represent its own subspecies (*O*. *mykiss aquilarum*; [25]). Testing against additional fish from this same hatchery strain (*n* = 5) found this C_t_ delay to be consistent across fish from the Eagle Lake strain, indicating the presence of an intraspecific polymorphism in one of the primers (confirmed by additional sequencing to be due to a polymorphism on the 3’ end of the reverse primer; *n* = 5 Eagle Lake individuals, S1 Text). Based on the standard curve experiments, this assay had an amplification efficiency of 95.9% (standard curve y-intercept = 40.7, *r* ^*2*^ = 0.998) and limit of detection of 10 mtDNA copies/rxn.

## Discussion

We developed taxon-specific eDNA markers for two subspecies of cutthroat trout and for rainbow trout. These markers did not result in cross-amplification among these three taxa or other congeneric (*Oncorhynchus*) or confamilial (*Salmonidae*) species, even when target DNA constituted a minute fraction of the DNA in laboratory mixtures. Using software that models PCR chemistry in conjunction with induced base-pair mismatches and primer-limiting reactions allowed us to distinguish sequences that differ at only a few loci between RBT, WCT, and YCT.

Our emphasis on obtaining samples from throughout most of their respective geographic ranges enabled us to design assays capable of detecting many populations of the target taxa. Nevertheless, we discovered intraspecific polymorphisms among populations of two taxa that reduced assay sensitivity and could lead to less consistent or failed detections in field samples (i.e. reduced intraspecific generality), and a lack of specificity in one assay that could lead to ambiguity with regard to which subspecies of cutthroat trout was present.

Our RBT marker efficiently amplified fish from multiple coastal rainbow trout (*O*. *mykiss irideus*) and Columbia redband trout/steelhead (*O*. *mykiss gairdneri*) strains. Reduced sensitivity (~2 C_t_ amplification curve delay) was only found in a single hatchery strain (Eagle Lake) that may represent its own subspecies [25]. This issue is expected when a single taxon (*O*. *mykiss*) includes multiple, divergent lineages (Fig 1A). In the case of WCT, the polymorphism in the Youngs Creek sample which prevented amplification of some individuals could not be predicted based on previous phylogeographic work [11,26]. It was only with a very large sample size (> 100 individuals sequenced or tested with the assay) that this polymorphism was found. The rarity of this polymorphism means that this assay is still broadly applicable across the range of WCT, but highlights what we suspect is a general problem: for broadly distributed taxa composed of several evolutionary lineages and represented by hundreds of populations, it may be difficult or impossible to develop a single eDNA assay that identifies all members of that taxon. It also suggests that some current assays may be of regional rather than global utility, or useful when confronted with limited mixtures of taxa, but not in other circumstances.

Broad-scale sampling prior to marker development, however, does not solve the issue of unresolved taxonomies that may influence both the ability of an assay to distinguish between closely related taxa and to have generality within a taxon. For example, YCT and BCT are recognized as separate subspecies [27], but their phylogenetic distinctiveness is ambiguous. Campbell et al. [18] and Loxterman & Keeley [11] were able to genetically separate these taxa only in portions of their ranges, and identified a third group (the southern BCT clade) as having diverged from Yellowstone and northern BCT ~ 1.0–1.6 million years ago. Perhaps unsurprisingly, our assay reliably detected eDNA from the YCT and northern BCT lineages (*n* = 19 and 6 individuals respectively). Individuals from Harkness Creek and the Glenwood Fish Hatchery represent individuals from the southern BCT clade, which displayed reduced amplification efficiency when screened with the YCT assay. This may indicate that there is insufficient phylogenetic divergence to distinguish two recognized taxa (Yellowstone and some lineages of BCT [20]; Fig 1B), and is also likely a case of grouping divergent lineages under a single recognized taxon (i.e., polyphyly; northern and southern clades within BCT; Fig 1C).

Although several recent eDNA studies have sampled broadly for marker development (e.g., [9]), we suspect the tradeoff between interspecific specificity and intraspecific generality has been under-appreciated when building assays to separate closely related sympatric species. The first step to addressing this issue lies in determining the range and frequency of intraspecific polymorphisms across populations of the target taxa. This is simply a restatement of a maxim of phylogeography: good phylogenetic assessments require comprehensive geographic sampling [28]. This type of broad sampling is more likely to produce truly general assays, and may reveal previously undetected, cryptic diversity. This truth has also been recognized for other molecular species identification tools (i.e. barcoding [10,29]). Moreover, assays need to be tested locally on collected tissue samples and, if possible, eDNA samples taken from areas with confirmed presence of the target species prior to application. Additionally, it may be useful to apply multiple markers at different loci to reduce the risk of false negatives (failure to detect a species when present) due to rare polymorphisms and to separate closely related species. Finally, eDNA studies have increasingly incorporated occupancy models that account for imperfect detection [30–32]. These are a useful tool for accounting for imperfect detection due to eDNA degradation and variation in eDNA capture, but may also reduce false negative inference due to rare polymorphisms that results in reduced detection probabilities.

## Supporting Information

S1 Text Sequencing information for westslope cutthroat trout and Eagle Lake rainbow trout. (DOCX)

S2 Text MIQE checklist for reporting qPCR assays. (XLS)
