# Supplementary material for: The Dual Challenges of Generality and Specificity When Developing Environmental DNA Markers for Species and Subspecies of Oncorhynchus
Source: PLoS One. 2015 Nov 4;10(11):e0142008. doi: 10.1371/journal.pone.0142008 (PMC4633235; doi:10.1371/journal.pone.0142008)
Supplement: S1 Text — (DOCX) [file pone.0142008.s001.docx]

**Text S1 for Developing environmental DNA markers for subspecies and species *of Oncorhynchus -*** Sequencing information for westslope cutthroat trout and Eagle Lake rainbow trout.

*Westslope cutthroat trout sequencing*

Ninety-six westslope cutthroat trout individuals were sampled from across the species’ range (Figure 2) under Montana Fish, Wildlife and Parks Scientific Collectors Permits 12-2001, 14-2001, and 19a-2009, U.S. Fish and Wildlife Service Federal Fish and Wildlife Permit TE220826-0 issued to MKY or was provided by collaborators who collected samples under the appropriate scientific collector’s permits. A fin clip from each individual was stored on chromatography paper or in ethanol in the field and then DNA was extracted in the lab using QIAGEN DNeasy Blood and Tissue Kit following the manufacturer’s protocol. Mitochondrial DNA was enriched using short oligonucleotide hybridization baits and sequenced on a HiSeq2000 (Illumina). Sequences were read mapped using BLAT. We extracted a 3,483 bp region of the mitogenome sequences including the *NADH* subunits 1 and 2 for assay design. Below are haplotypes at each of the three final hydrolysis assays (WCT, RBT, and YCT; amplicon region including forward and reverse primers). All sequences are written 5’ to 3’.

>WCTmarker_hap1

CCTAAAACTATTTATTAAAGAACCAGTTCGACCTTCCACCTCCTCTCCCTTTCTATTTCTCGCTACCCCTATACTCGCCCTTACACTT

>WCTmarker_hap2

CCTAAAACTATTTATTAAAGAACCAGTTCGACCTTCCACCTCCTCTCCCTTTCTATTTCTCGCCACCCCTATACTCGCCCTTACACTT

>WCTmarker_hap3

CCTAAAACTATTTATTAAAGAACCAGTTCGACCTTCCAACTCCTCTCCCTTTCTATTTCTCGCTACCCCTATACTCGCCCTTACACTT

>WCTmarker_hap4

CCTAAAACTATTTATTAAAGAACCAGTTCGACCTTCCACCTCCTCTCCCTTTCTATTTCTCGCCACCCCCATACTCGCCCTTACACTT

>WCTmarker_hap5

CCTAAAACTATTTATTAAAGAACCAGTTCGACCTTCCACCTCCTCTCCCTTTCTATTTCTCACCACCCCTATACTCGCCCTTACACTT

>RBTmarker_hap1

AGCCTATCCCTATATATTATCATAACATCTTCAGCATTCCTCACATTAAAAACCAACAACGCTTTAACCATTAACACTCTCGCGACTTCATGAACTAAATC

>YCTmarker_hap1

CGACCTTCCACCTCCTCTCCCTTTCTATTTCTCGCTACCCCTATACTCGCCCTTACACTTGCGCTCACTCTATGGGCCCCCATACCTATTCCTTACCCTGTCACAGATCTTAACCTTGGAGTACTATTTGTGCTTGCACTATCCAGCCTAGCT

>YCTmarker_hap2

CGACCTTCCACCTCCTCTCCCTTTCTATTTCTCGCCACCCCTATACTCGCCCTTACACTTGCGCTCACTCTATGGGCCCCCATACCTATTCCTTACCCTGTCACAGATCTTAACCTTGGAGTACTATTTATGCTTGCACTATCCAGCCTAGCT

>YCTmarker_hap3

CGACCTTCCACCTCCTCTCCCTTTCTATTTCTCGCCACCCCTATACTCGCCCTTACACTTGCGCTCACTCTATGGGCCCCCATACCTATTCCTTACCCTGTCACAGATCTTAACCTTGGAGTACTATTTGTGCTTGCACTATCCAGCCTAGCT

>YCTmarker_hap4

CGACCTTCCACCTCCTCTCCCTTTCTATTTCTCGCCACCCCCATACTCGCCCTTACACTTGCGCTCACTCTATGGGCCCCCATACCTATTCCTTACCCTGTCACAGATCTTAACCTTGGAGTACTATTTGTGCTTGCACTATCCAGCCTAGCT

>YCTmarker_hap5

CGACCTTCCACCTCCTCTCCCTTTCTATTTCTCGCTACCCCTATACTCGCCCTTACACTTGCGCTCACTCTATGGGCCCCCATACCTATTCCTTACCCTGTTACAGATCTTAACCTTGGAGTACTATTTGTGCTTGCACTATCCAGCCTAGCT

>YCTmarker_hap6

CGACCTTCCAACTCCTCTCCCTTTCTATTTCTCGCTACCCCTATACTCGCCCTTACACTTGCGCTCACTCTATGGGCCCCCATACCTATTCCTTACCCTGTCACAGATCTTAACCTTGGAGTACTATTTGTGCTTGCACTATCCAGCCTAGCT

>YCTmarker_hap7

CGACCTTCCACCTCCTCTCCCTTTCTATTTCTCGCTACCCCTATACTCGCCCTTACACTTGCGCTCACTCTATGGGCCCCCATACCTATTCCTTACCCTGTCACAGATCTTAACCTTGGAGTACTATTTGTGCTTGCACTGTCCAGCCTAGCT

*Eagle Lake rainbow trout sequencing*

To determine the polymorphism causing reduced amplification efficiency of fish from Eagle Lake, we sequenced the amplicon region of this assay for five individuals. These were amplified using primers 5’- ATTCGCACCCTCTCTCACAC-3’ and 5’- GGAGACCTCCAAGGGACAAT-3’ and 50 – 100 ng of tissue-extracted DNA on a StepOne Plus instrument (Life Technologies) following the protocol for SYBR Green reactions listed in the manuscript. PCR products were purified using ExoSap-IT (Affymetrix-USB Corporation) following the manufacturer’s protocol. DNA sequence data was obtained using the Big Dye kit and a 3700 DNA Analyzer (ABI; High Throughput Genomics Unit, Seattle, Washington, USA) using the same primers as for amplification. All primers were ordered from Integrated DNA Technologies. All five fish had the same haplotype, which is listed below. Sequence is written 5’ to 3’.

>RBT_NADH_EagleLake_hap1

ATTCGCACCCTCTCTCACACTCCTCAGTCTCTCCCTGTATATCGTCATAACATCTTCAGCTTTCCTCACATTAAAAACCAACAACTCTTTAACCATCAACACTCTCGCAACTTCATGAACTAAATC
